# Infection dynamics and tissue tropism of *Parvicapsula pseudobranchicola* (Myxozoa: Myxosporea) in farmed Atlantic salmon (*Salmo salar*)

**DOI:** 10.1186/s13071-017-2583-9

**Published:** 2018-01-06

**Authors:** Are Nylund, Haakon Hansen, Øyvind J. Brevik, Håvard Hustoft, Turhan Markussen, Heidrun Plarre, Egil Karlsbakk

**Affiliations:** 1 Department of Biology, University of Bergen, 5020 Bergen, Norway; 2 Norwegian Veterinary Institute, PO Box 750 Sentrum, N-0106 Oslo, Norway; 3 Cermaq group AS, Dronning Eufemias gate16, P.O. Box 144, N-0102 Oslo, Norway; 4 Faculty of Veterinary Medicine, Norwegian University of Life Sciences, Oslo, Norway; 5 Institute of Marine Research, PO Box 1870, Nordnes, N-5817 Bergen, Norway

**Keywords:** Parasites, *Salmo salar*, Aquaculture, Disease, Norway, *in situ* hybridization, Real-time RT-PCR

## Abstract

**Background:**

The myxosporean parasite *Parvicapsula pseudobranchicola* commonly infects farmed Atlantic salmon in northern Norway. Heavy infections are associated with pseudobranch lesions, runting and mortality in the salmon populations. The life-cycle of the parasite is unknown, preventing controlled challenge experiments. The infection dynamics, duration of sporogony, tissue tropism and ability to develop immunity to the parasite in farmed Atlantic salmon is poorly known. We conducted a field experiment, aiming at examining these aspects.

**Methods:**

Infections in a group of Atlantic salmon were followed from before sea-transfer to the end of the production (604 days). Samples from a range of tissues/sites were analysed using real-time RT-PCR and histology, including *in situ* hybridization.

**Results:**

All salmon in the studied population rapidly became infected with *P. pseudobranchicola* after sea-transfer *medio* August. Parasite densities in the pseudobranchs peaked in winter (November-January), and decreased markedly to March. Densities thereafter decreased further. Parasite densities in other tissues were low. Parasite stages were initially found to be intravascular in the pseudobranch, but occurred extravascular in the pseudobranch tissue at 3 months post-sea-transfer. Mature spores appeared in the pseudobranchs in the period with high parasite densities in the winter (late November-January), and were released (i.e. disappeared from the fish) in the period January-March. Clinical signs of parvicapsulosis (December-early February) were associated with high parasite densities and inflammation in the pseudobranchs. No evidence for reinfection was seen the second autumn in sea.

**Conclusions:**

The main site of the parasite in Atlantic salmon is the pseudobranchs. Blood stages occur, but parasite proliferation is primarily associated with extravascular stages in the pseudobranchs. Disease and mortality (parvicapsulosis) coincide with the completion of sporogony. Atlantic salmon appears to develop immunity to *P. pseudobranchicola*. Further studies should focus on the unknown life-cycle of the parasite, and the pathophysiological effects of the pseudobranch infection that also could affect the eyes and vision.

**Electronic supplementary material:**

The online version of this article (10.1186/s13071-017-2583-9) contains supplementary material, which is available to authorized users.

## Background

Many myxosporeans are important disease agents, causing tissue damage and mortality in farmed fish [1]. The myxosporean *Parvicapsula pseudobranchicola* (Parvicapsulidae) was originally described from diseased farmed Atlantic salmon in Norway, where it was found to infect the pseudobranchs [2]. The parasite has later been found to infect other salmonids in the northeast Atlantic [3–5], and certain *Oncorhynchus* spp. in the eastern Pacific (British Columbia) [6, 7]. However, several of these records represent molecular detections, mature spores of the parasite have so far only been observed in wild and farmed Atlantic salmon, farmed rainbow trout and wild seatrout in Norway [4, 5, 8, 9].

The life-cycle of *P. pseudobranchicola* is unknown. Myxosporeans show two-host life-cycles, involving a vertebrate host where myxospores are produced, and an invertebrate host where development culminates in the production of actinospores. All currently known alternate invertebrate hosts are annelids [10, 11]. Parvicapsulid life-cycles have been found to involve polychaetes from several families within the order Sabellida, where actinospores of the tetractinomyxon type are produced [12–14]. While present throughout Norway, *P. pseudobranchicola* infections in seawater farmed salmon are particularly frequent and heavy in the northern counties [3, 8, 15], and autumn stocked salmon generally show a 100% prevalence within two to four weeks [9]. A high infection pressure seems to be present from late summer to early winter [9], and may be due to high actinospore densities in the sea. The port of entry and early development in salmon is poorly known. Molecular evidence has suggested that initial blood stages may occur [8], as in some other myxosporeans (e.g. [16–18]). Sporogonic stages and spores primarily occur in the pseudobranchs, but have occasionally been detected in other organs in farmed Atlantic salmon, such as the gills, kidney and the liver [19].

Individuals with heavy infections have been observed to surface, swim disorganized or appear lethargic, and may be unresponsive to visual challenge as if blind. The eyes usually show crescent shaped hemorrhaging, and cataracts and exophthalmia may also occur. The fish do not feed and tend to be slim and anaemic [2, 19]. Affected pseudobranchs may be swollen or papillate, in severe cases they may show whitish coverings occasionally hemorrhaging. Pseudobranchs may also be more or less replaced by ulcers [2, 3, 20]. Histologically, sporogonic stages occur intracellularly in the pseudobranch cells, while mature spores may occur free or in pseudoplasmodia in a necrotic debris, filling the space between the pseudobranch secondary lamellar lacunae. Since the blood supply to the eyes passes via the pseudobranchs, it has been suggested that massive infections by the parasite and the tissue destruction accompanying sporogony may affect the blood supply and cause blindness [2, 19]. Such an interpretation is in accordance with the clinical signs connected with heavy infections [2]. However, since the function of the pseudobranch is poorly known [21, 22], the pathophysiological effects of pseudobranch-destruction remain unclear. Losses ascribed to parvicapsulosis are due to both mortality and culling, and in some cohorts may reach 35%. Hence, this myxosporean may have a high impact on the salmon farming in the northern parts of Norway.

In pseudobranchs of Atlantic salmon, mature *P. pseudobranchicola* spores have been observed some 4–8 months after sea-transfer [2, 8, 19, 23]. However, the time needed for the parasite to develop spores is poorly known, but nonetheless relevant because clinical parvicapsulosis may relate to inflammation associated with the completion of sporogony [5]. Although *P. pseudobranchicola* sporogony has been observed in different organs, the relative contribution of these to the total spore production is unknown. Even though high spore densities occur in the pseudobranchs, this organ is small in comparison to the gills, liver and kidneys where spore production has also been detected [19]. Most parvicapsulids are kidney parasites, releasing spores via urine, and a significant contribution of the kidney to the total spore output for *P. pseudobranchicola* seems possible. In the present study, we followed a cohort of farmed Atlantic salmon throughout a marine production cycle of 601 days providing new information on the infection dynamics, development, tissue tropism and the risk of re-infection.

## Methods

### The farm and study-population

Farmed Atlantic salmon, *Salmo salar* (*n* = 1.04 million) kept at a marine site near Sørøya (70°62′N, 23°10′E) in Finnmark County, northern Norway, were followed during a production period from August 2014 until commercial size was reached in May 2016. The production site is located in Sørøysundet, a large strait with strong currents. The bottom under the farm is sloping, the depth ranging between 60 and 120 m.

Different smolt groups were sea launched from late July to late September 2014. The smolts originated from three hatcheries located in Nordland County, and had been transported for 3 days in well-boats to reach the farm site. The present study focused on fish from a single cage (No. 4) of the 10 present, receiving smolts sea-launched the 14th of August 2014 (*n* = 116,850). Mortality and seawater temperature (at 2 m depth) were recorded daily. Tenacibaculosis due to *Tenacibaculum finnmarkense* affected the farm during the two first months at sea, resulting in culling of all fish in a neighbouring cage (No. 9), the most severely affected group. All other fish received a 10-day standard treatment with florfenicol, which arrested the mortality. However, this incident resulted in a peak in mortality in the farm as well as in the study cage in week 36. A second period with elevated mortality occurred from week 48 in 2014 to week five 2015.

### Samples

Samples were first taken from smolts at the freshwater site (*n* = 25 fish), the day before being transported to the marine location, and then ten times during the marine production phase (Table 1). Nine of the sample times represented regular samples (*n* = 30), while one was additional (day 106, see below). The first seven samples from the seawater pens were collected at the site while the last three samples were taken from fish (head-ends) sent freshly frozen to the laboratory at the University of Bergen. These had been cut behind the pectorals, so length and weight data were not taken.

**Table 1:** Overview of sample dates, days post-sea-transfer, number of fish sampled (*n*), tissue samples taken and the analyses performed

Dates	Days	*n*	Tissues	Analyses
2014				
11.08.	-3	25	P, H	PCR, ISH.
04.09.	21	30	P, E, G, H, K, L, I, S, B	PCR, Hi, ISH
18.09.	35	30	P, E, G, H, K, L, I, S, B	PCR, Hi, ISH
02.10.	49	30	P, E, G, Oe, H, K, L, I, S, B	PCR, Hi, ISH
11.11.	89	30	P, E, G, H, K, L, I, S, B, Gb, U	PCR, Hi, ISH, M
28.11.	106	5^a^	P	M
2015				
08.01.	147	30	P, E, G, H, K, L, S, B, Gb, U	PCR, Hi, ISH, M
18.03.	216	30	P, E, G, H, K, L, S, B, Gb, U	PCR, Hi, ISH, M
27.08.	347	30	P, E, G, H, K	PCR
08.12.	451	30	P, G, H, K	PCR
2016				
06.05.	601	30	P, G, H, K	PCR

*Abbreviations*: *P* pseudobranch, *E* eye, *G* gills, *Oe* oesophagus, *H* heart, *I* intestine, *K* kidney, *S* spleen, *L* liver, *Gb* gall bladder, *U* urinary bladder, *PCR* real-time RT-PCR, *Hi* histology, *ISH* *in situ* hybridization, *M* microscopy on pseudobranch squash preparations

^a^Additional sample of fish selected based on clinical signs, examined for spore development

Some fish were separated from the pen-population using a small closing-net raised from below when hand feeding the fish. The fish in this group were then crowded, before removing fish for sampling at random with a landing net. The fish caught for sampling at the site were kept alive in a temporary fish tank (1.0 m^3^) with continuous water flow. Dissection of each fish was done immediately after killing the fish. The fish were killed with a blow to the head, except those sampled for histology, which were killed with an overdose anaesthetic (Benzoak® vet, ACD Pharmaceuticals AS, Leknes, Norway). Fork length (L, cm) and weight (W, g) of each fish were measured. Blood samples were taken from the caudal vessels using 1 ml syringes (disposable, no anticoagulant), and blood smears made. All fish were examined for macroscopical lesions and signs of disease. Internal organs/tissues were aseptically dissected from each fish. Samples collected for real-time RT-PCR were kept on 70% ethanol during field work and transferred to 100% ethanol at the laboratory and stored at -20 °C.

Samples for real-time RT-PCR were taken from 11 organs/sites in the fish, with the 4 organs pseudobranch, gill (2nd arch), mid-kidney and heart (ventricle) being sampled throughout in the marine phase (Table 1). Blood, spleen and liver samples were taken from freshly examined fish (first 7 months), while eye samples (targeting choroidea) were taken the first year. In the period when spores were detected in the pseudobranchs, samples were also taken from bile, the gallbladder and the urinary bladder. This was done to reveal molecular evidence for spore release, since our small liver and kidney samples could miss infection foci in these large organs. Samples from the intestine were taken initially, but discontinued due to very weak signals in real-time RT-PCR analyses. However, due to the continuous drinking of seawater by teleosts in the marine environment, a single sample of the oesophagus was taken in October, since this site represents a potential port of entry for the parasite.

### Histology and *in situ* hybridization (ISH)

Tissues for histological examination were fixed in formalin and transferred to 70% ethanol after 24 h and to 100% ethanol after 48 h and stored at 4 °C. These were embedded in paraffin, sectioned (3–5 μm), and mounted onto Superfrost™ Plus glass slides (Thermo Scientific, Braunschweig, Germany). For each tissue section destined for ISH, neighboring sections were also collected and mounted. The additional sections were stained with hematoxylin and eosin (HE), enabling direct comparisons between ISH and HE stained sections. The ISH procedure followed Markussen et al. [24], with one modification. Due to the expected higher endogenous enzymatic activities present in some of the tissue types investigated, neutralization was performed by incubation in 1% H_2_O_2_ (Sigma-Aldrich, St-Louis, MO) as opposed to the 0.1% used in the previous studies on pseudobranch tissue [5, 24]. ISH was performed on tissues from two of the five fish sampled for this purpose at each sample date; providing a suitable parasite density. The selection was based on the real-time RT-PCR results.

### RNA extraction and real-time RT-PCR

RNA was extracted from the sampled tissue as described by Gunnarson et al. [25]. To increase the quality of the RNA, an additional washing step using 96% ethanol was performed. The RNA pellet was finally eluted in 50 μl RNase-free water preheated to 70 °C. *Prior* to RNA extraction from blood, the samples were vortexed a few seconds and 200 μl transferred to new tubes. After centrifugation of the samples for 5 min at 13,400× *g*, the ethanol was pipetted out and the RNA extracted as described above. The purity and concentration of RNA was tested using a NanoDrop ND-1000TM spectrophotometer.

Real-time RT-PCR analyses were performed using AgPath-ID™ One-Step RT-PCR Kits (Applied Biosystems, Austin, Texas). Assays targeting *P. pseudobranchicola* (Parvi) and the elongation factor 1 alpha were used throughout (Table 2). In addition, samples from the smolts before transfer to sea and the salmon collected at the marine site 147 and 601 days after sea transfer were tested for other pathogens known to be present in farmed Atlantic salmon in the region (see Table 2 for details on targets and the assays used). The RT-PCR was run in a total volume of 12.5 μl using 2 μl of RNA sample on a 7500 Real-time PCR System and a 7500 Fast Real time PCR System cycler (Applied Biosystems). Cycling conditions were 45 °C/10 min (RT step) and 95 °C/10 min followed by 45 cycles of 95 °C/15 s and 60 °C/45 s. The concentration of primers (10 μM) and probe (10 μM) had been optimized for these assays.

**Table 2:** Real time RT-PCR assays used in the study

Target (Assay name)	Primer and probe sequences (5′–3′)		Reference
Atlantic salmon Elongation factor 1α (EF1A_A_)	F	CCCCTCCAGGACGTTTACAAA	[40]
	Probe	ATCGGTGGTATTGGAAC	
	R	CACACGGCCCACAGGTACA	
*Parvicapsula pseudobranchicola* 18S (Parvi)	F	TCGTAGTCGGATGACAAGAACGT	[29]
	Probe	CCGTATTGCTGTCTTTGA	
	R	AAACACCCCGCACTGCAT	
*Desmozoon lepeophtherii* 16S (Nuc*)*	F	CGGACAGGGAGCATGGTATAG	[41]
	Probe	TTGGCGAAGAATGAAA	
	R	GGTCCAGGTTGGGTCTTGAG	
*Ichthyobodo* spp. 18S (Costia)	F	ACGAACTTATGCGAAGGCA	[42]
	Probe	TCCACGACTGCAAACGATGACG	
	R	TGAGTATTCACTYCCGATCCAT	
“*Candidatus* Branchiomonas cysticola” 16S (Epit)	F	GAGTAATACATCGGAACGTGTCTAGTG	[43]
	Probe	ACTTAGCGAAAGTTAAGC	
	R	CTTTCCTCTCCCAAGCTTATGC	
Piscine orthoreovirus M2 (PRV)	F	CAATCGCAAGGTCTGATGCA	[43]
	Probe	CTGGCTCAACTCTC	
	R	GGGTTCTGTGCTGGAGATGAG	
Piscine myocarditis virus (PMCV)	F	AGGGAACAGGAGGAAGCAGAA	[43]
	Probe	TGGTGGAGCGTTCAA	
	R	CGTAATCCGACATCATTTTGTGA	
Infectious pancreatic necrosis virus Segm. A (IPNV)	F	ACCCCAGGGTCTCCAGTC	[29]
	Probe	TCTTGGCCCCGTTCATT	
	R	GGATGGGAGGTCGATCTCGTA	
Infectious salmon anemia virus S7 (ISAV)	F	TGGGATCATGTGTTTCCTGCTA	[44]
	Probe	CACATGACCCCTCGTC	
	R	GAAAATCCATGTTCTCAGATGCAA	

*Abbreviations*: *F* forward, *R* reverse

Efficiency (E) and Ct-values of the different assays were used when calculating the normalized expression (NE) of the target using the EF1A_A_ as a reference gene: NE = (E_ref_)^Ct ref^/ (E_target_)^Ct target^ [26].

### Pseudobranch squash preparations

In addition to histological examination, direct microscopy was performed on pseudobranch squash preparations from some samples. This was done in order to examine the progression of sporogony, since the maturity of the spores may be difficult to evaluate based on histology alone. Direct microscopy was performed on material collected at days 89, 106, 147 and 216 post-sea-transfer.

### Statistical analyses

Normalized expression (NE) of *P. pseudobranchicola* small-subunit (SSU) rRNA was used as a measure of parasite density, and in the statistical analyses as a proxy of parasite abundance (for quantitative terms, see [27]). NE in negative samples was set at ‘0’. The NE data were usually heteroscedastic and non-normal. Therefore, the temporal changes in parasite density were examined with the non-parametric Kruskal-Wallis (K-W) ANOVA by ranks. Significant steps in significant ANOVA’s were identified with the multiple comparisons (MC) tests accompanying K-W. Concordance in mean parasite densities across tissues was examined using Kendall’s coefficient of concordance (*W*) [28], using samples from days 21–216 after sea-transfer. Prevalence was compared using Fisher’s exact tests (FET). Kendall’s *W* was calculated in a Microsoft Excel spreadsheet. All other statistical tests were performed with Statistica 64 (Dell Inc., Tulsa, USA).

## Results

### Mortality, clinical observations and other infections

**Fig. 1:**
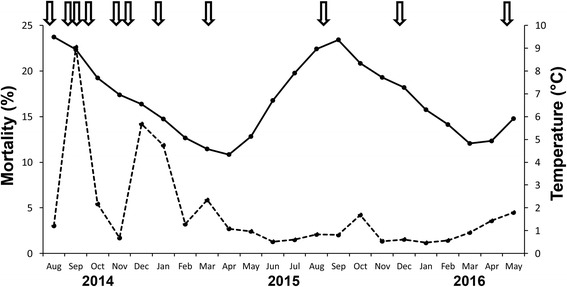
Overview of monthly mortality and sea water temperature during the sampling period. The dashed line shows the monthly mortality in the study-cage (No. 4), as a % of the total production cycle mortality in the cage. The continuous line shows the monthly mean temperature. The first peak in mortality (September) was due to a tenacibaculosis outbreak, while the second peak (December-January) was associated with high *Parvicapsula pseudobranchicola* intensities in the pseudobranchs. Arrows at top indicate when the presently studied samples were taken

The average seawater temperature (2 m depth) ranged from 4 °C (March-April) to 10 °C (August-September) during the production period (Fig. 1). A slight increase in mortality in the study cage occurred during a tenacibaculosis outbreak starting 20 days post-sea-transfer (PST). Both the mortality and morbidity declined after an antibiotic treatment in week 38 and levelled at normal mortality in week 43. From sea transfer to the end of the production period the total accumulated mortality in the study cage was 10.5%, of which some 2.9% was associated with the tenacibaculosis outbreak (first peak in Fig. 1) and about 3% with a second peak in mortality in winter (Fig. 1). These peaks represented about 28% and 29%, respectively, of the total production cycle mortality in the pen.

At each sampling date PST, gross pathology was registered. At 35 days PST during the tenacibaculosis outbreak, some of the fish had skin ulcers in the head region and occasionally on the lateral sides and in the fins. The pseudobranchs were normal, except that two fish showed protruding swellings on their pseudobranchs. At 49 days PST, there was still elevated mortality due to the tenacibaculosis in the study cage, and five of the collected salmon showed such swellings in the pseudobranchs. The fish exhibited a decrease in appetite 89 days PST, and then 33% of the fish showed pseudobranch lesions, six fish with swellings and four with whitish coverings. At the fifth sampling (147 days PST) there was elevated mortality at the site, coinciding with a storm. Some fish surfaced, appeared lethargic and swam in a disorganized fashion. Macroscopically, most pseudobranchs were covered with a whitish matter. Fish sampled 216 days PST and later in the production period showed no pseudobranch changes or signs of disease.

The Atlantic salmon population followed in this study were tested (real time RT-PCR) for a range of regionally relevant pathogens at three different time points during the production: (i) smolt stage in fresh water; (ii) at peak winter mortality 147 days PST; and (iii) at the termination of the production (601 days PST). The smolt were negative for presence of *P. pseudobranchicola*, PRV, ISAV and PMCV before sea transfer, but 9 (*n* = 25) fish were positive for IPNV (Ct 34.2–36.0). All salmon collected at 147 days PST were negative for presence of piscine orthoreovirus (PRV), infectious salmon anemia virus (ISAV), piscine myocarditis virus (PMCV), infectious pancreatic necrosis virus (IPNV), “*Candidatus* Branchiomonas cysticola” and *Ichthyobodo salmonis*. At the termination of the production, all salmon were positive for PRV, a few were positive for PMCV, ISAV (HPR0) and *I. salmonis.* All fish were then negative for IPNV.

### Prevalence of infection

A total of 294 Atlantic salmon were screened for the presence of *P. pseudobranchicola* during the study period (14th August 2014 to 5th May 2016) using real-time RT-PCR. The smolt sampled before sea transfer (14th August) were negative for the parasite. Already in the first sample 21 days PST (4th September), the prevalence had reached 100% (based on analyses of the pseudobranchs). Prevalence stayed at 100% in the samples from the first year at sea. At 451 and 601 days PST, a few negative fish were registered (Table 3.).

**Table 3:** Prevalence of *Parvicapsula pseudobranchicola* RNA presence in four different tissues collected during the study period of 601 days post sea-transfer

Date	dpst	Ps	Gi	Ki	He	Bl	Li	Sp	In	Ey	Ub	Gb
4 Sep.	21	100	93	100	67	57	60	97	10	97	–	–
18 Sep.	35	100	97	100	97	100	100	100	67	97	–	–
2 Oct.	49^a^	100	100_*11*_^b^	100	100_*12*_	100	100_*12*_	92_*12*_	100_*12*_	100_*12*_	–	–
11 Nov.	89	100	100	100	100_*5*_	100	100_*5*_	100_*5*_	100_*5*_	100_*5*_	100_*5*_	86_*7*_
08 Jan.	147	100	100	100	100_*5*_	100	100_*6*_	100_*5*_	–	100_*5*_	100_*5*_	80_*5*_
18 Mar.	216	100	100	100	100	97	97	100	–	97	100	60
27 Aug.	347	100	100	97	100	–	–	–	–	83	–	–
08 Dec.	451	97	80	57	33	–	–	–	–	–	–	–
6 May.	601	90	90	73	57	–	–	–	–	–	–	–

*Abbreviations*: *Date* collection date, *dpst* days post-sea-transfer, *Ps* pseudobranch, *Gi* gills, *Ki* kidney, *He* heart, *Bl* blood, *Li* liver, *Sp* spleen, *In* intestine, *Ey* eye, *Ub* urinary bladder, *Gb* gall bladder, *−* not taken

^a^In this sample also oesophagus, 100% (*n* = 12)

^b^Reduced sample sizes (*n*) compared with Table 1 given as subscripts

The parasite RNA was detected in all tissues tested with the lowest prevalence observed in the gut wall (10 and 67%) days 21–35 PST. Blood was positive for the parasite in all samples, but prevalence in the first sample taken after sea transfer (day 21) was only 57%. Thereafter, prevalence of the parasite in blood was 100% through January 2015. At 35 days PST the prevalence in the other tissues had increased to about 100% (Table 3). The prevalence of *P. pseudobranchicola* RNA positive tissues remained high throughout the first year, ranging from 97 (kidney) to 100% (pseudobranch, gills, and heart) after 347 days. Samples from the eye showed a high prevalence through the first winter, being significantly higher than in the blood at 21 days PST (FET, *P* < 0.001). Gall- and urinary bladder samples were taken at days 89–216 only. Prevalence was 86% and 60% in these gallbladder samples, respectively, and 100% in the urinary bladder samples.

### Parasite densities

**Fig. 2:**
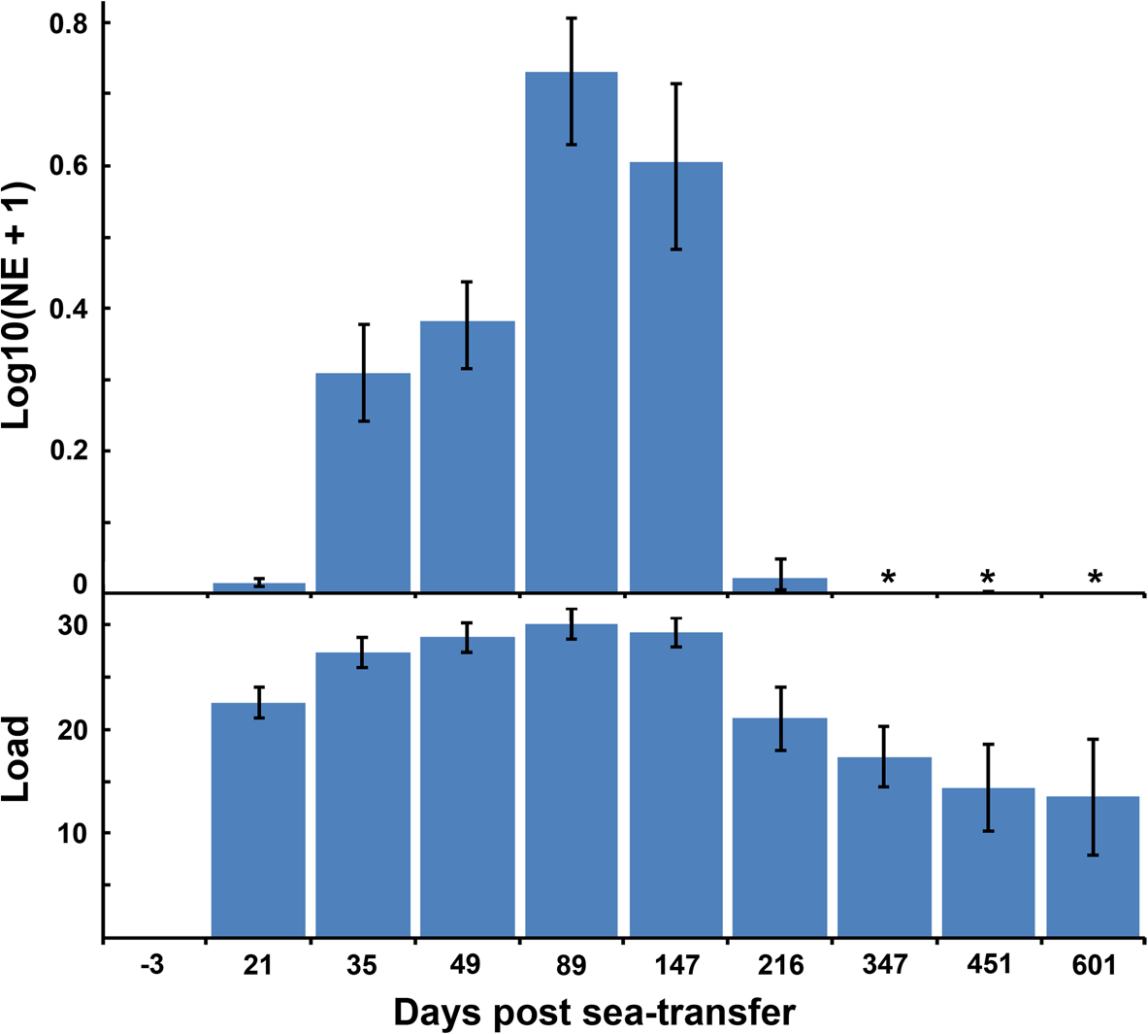
Density of *Parvicapsula pseudobranchicola* in the pseudobranchs of Atlantic salmon collected during the study period of 604 days, estimated as normalized expression (NE) of small subunit rRNA or ‘load’ (*n* cycles = 45-Ct). The first sample (-3) was taken prior to sea-transfer, when all fish were uninfected. Error bars for NE represent bootstrapped 95% confidence intervals, for ‘load’ they represent standard deviations. Asterisks (*): very low mean NE values; columns present but invisible (mean NE < 0.01)

Real time RT-PCR analyses revealed *P. pseudobranchicola* RNA to be present in all sampled tissues throughout the sampling period PST. The density of the parasite in the pseudobranch varied significantly (Kruskal-Wallis (K-W), *H*_(9, *N* = 294)_ = 264.6, *P* < 0.001), A gradual increase occurred from sea transfer to a high-level 89–147 days PST (November-January) (Fig. 2) (K-W, MC, *P* < 0.001). NE decreased markedly (K-W, MC, *P* < 0.001) from the 8th January (day 147) to the 18th March sample (216 days PST). Through the rest of the production period *P. pseudobranchicola* RNA densities in the pseudobranchs were low, with a gradual but significant (K-W, MC, *P* < 0.001) further decrease to day 601 PST (Fig. 2).

**Fig. 3:**
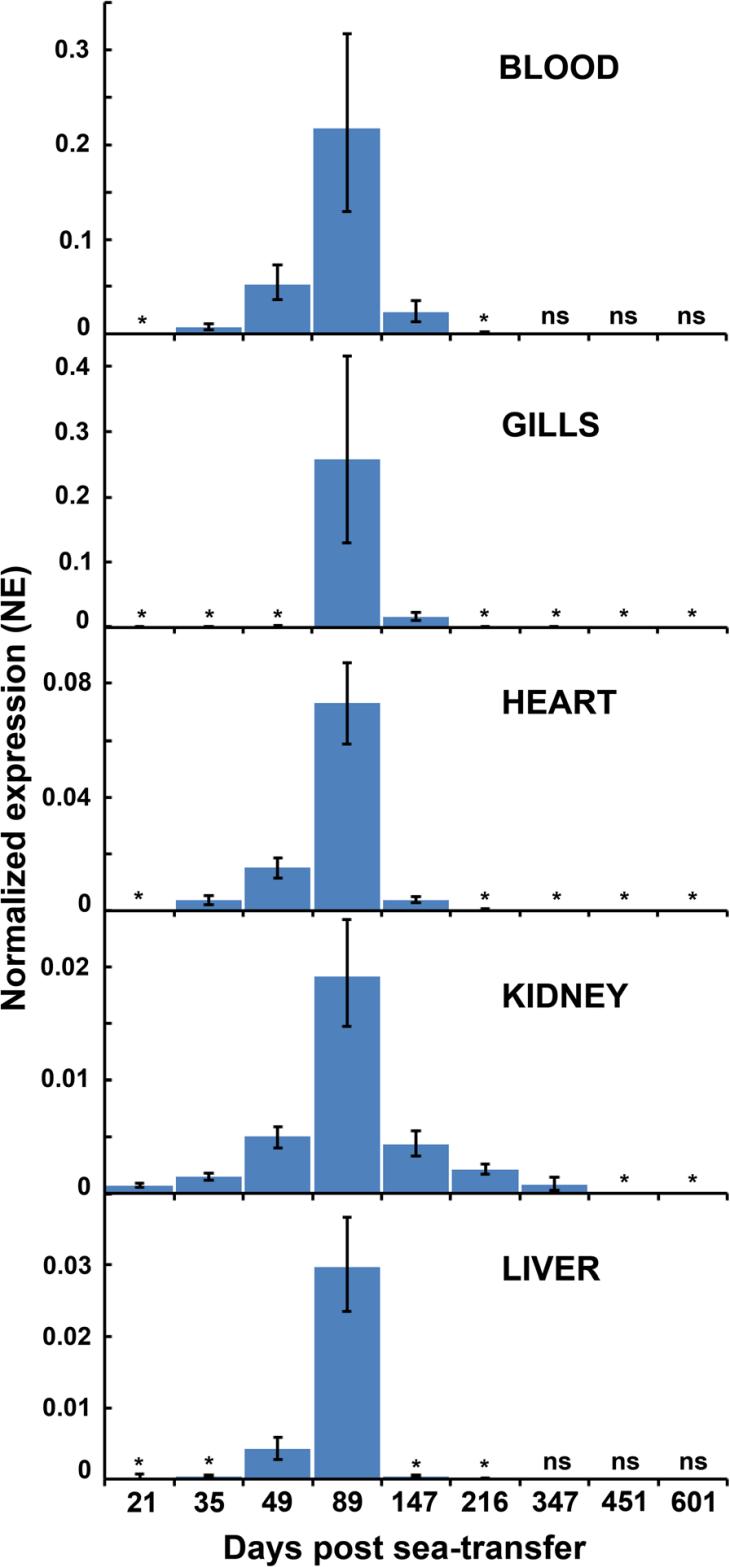
Mean normalized expression (MNE) of *Parvicapsula pseudobranchicola* small subunit rRNA in samples from blood, gills, heart, kidney and liver of Atlantic salmon collected during the study period. MNE is highly concordant days 21–216 (all samples), and days 21–601 (gills, heart, kidney). Note that the vertical scales differ, and that the MNE values are not directly comparable across tissues. Asterisks (*): very low NE; columns present but invisible, ‘ns’, no sample

Compared to other tissues/organs, the parasite density was highest in the pseudobranchs throughout. Parasite density in the blood was low, but varied significantly with an increase the first 49 days (K-W, MC, *P* < 0.001), followed by relatively high level days 49–147 (K-W, MC, *P* > 0.05) and a significant drop (K-W, MC, *P* < 0.001) to day 216 (Fig. 3a). The gills, heart, kidneys and liver showed a similar pattern in the parasite densities to the blood, and mean NE (MNE) in these five sets of samples were highly concordant (W_(k = 5,*N* = 6)_ = 0.80, *P* < 0.001) (Fig. 3). NE in spleen, eye and intestine also followed this pattern. The NE of *P. pseudobranchicola* rRNA in intestine, oesophagus, gall bladder, spleen and urinary bladder were low in all samples, usually much lower than in blood.

NE of the parasite in the pseudobranch and blood samples were not correlated in any sample. However, NE in gills and pseudobranchs showed a clear positive correlation (overall *r*_s_ = 0.80, *n* = 248; *P* < 0.001); when examined in the different samples this correlation was found to be strongest at high *P. pseudobranchicola* density at day 147 (*r*_s_ = 0.68, *n* = 30; *P* < 0.001).

Parasite density in the pseudobranch did not correlate significantly with host condition (W/L^3^) in any sample.

The dataset is provided in Additional file 1: Table S1.

### Histology and *in situ* hybridization

**Fig. 4:**
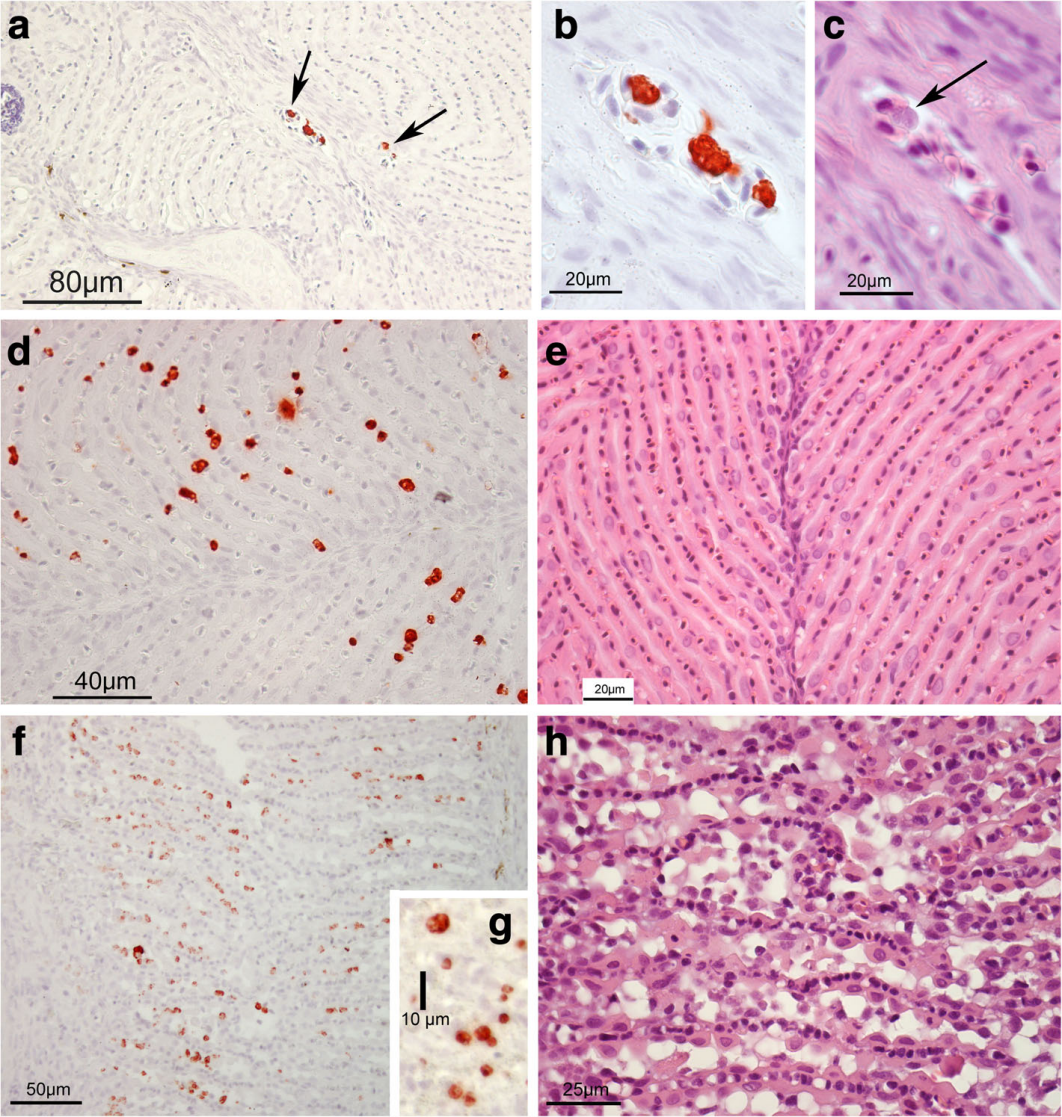
*Parvicapsula pseudobranchicola* infection in the pseudobranchs of Atlantic salmon, in histological sections stained using *in situ* hybridization (ISH) or hematoxylin-eosin (HE). **a**-**c** At 35 days post-sea-transfer (PST) most parasite stages occurred inside the primary filament vessels (**a**, ISH, arrows). The parasite in **c** (HE) is in an adjacent section to those in **b** (ISH). **d**, **e** At 49 days PST, most parasite cells occur in the lamellar lacunae (**d**; ISH), but the tissues appear normal (**e**, HE). **f**. 89 days PST, when all parasites were extravascular, but small (ISH). **g** Detail showing parasite cells (ISH); **h** Accompanying tissue destruction; pseudobranch cell depletion and high cellularity due to numerous parasite cells (HE)

**Fig. 5:**
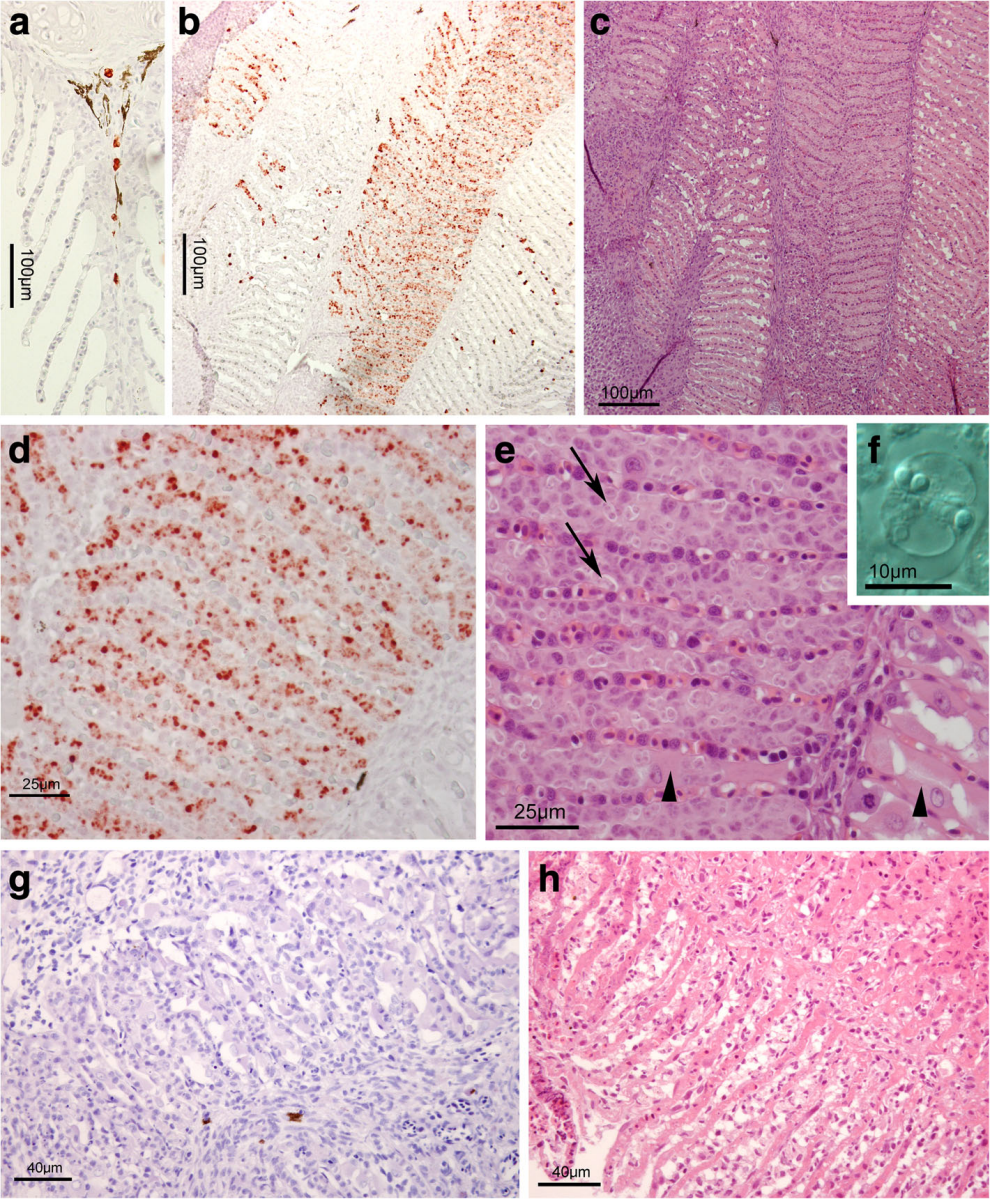
*Parvicapsula pseudobranchicola* infection in the gills (**a**) and pseudobranchs (**b**-**h**) of Atlantic salmon. **a** Gill primary filament day 89 PST with parasite stages (*red*) in blood vessel. **b**-**f** pseudobranch day 147 PST. **b** Overview (ISH) showing a compartmentalized occurrence of parasite stages; some areas between primary lamellae harbouring high parasite densities. **c** same in adjacent section (HE). **d** Detail of **b**, showing ISH stained parasites between the secondary lamellar lacunae. **e** Same pseudobranch as in **d**, arrows indicate nearly mature spores, and arrowheads intact pseudobranch cells. **f** Spores from wet preparation, at day 147 mature and immature spores occurred alongside earlier developmental stages. **g**-**h** Day 216 PST. **g** Pseudobranch with secondary lamellae, showing an irregular structure and high cellularity, but no parasite stages (ISH). **h** Same fish, other area (HE), showing tissue destruction

Using *in situ* hybridization (ISH), stained parasites were observed in the pseudobranch at days 35, 49, 89 and 147, and in the gills at day 89 PST. All other samples were negative. The ISH stained parasites were observed at two sites in the pseudobranch tissue, intravascular and extravascular. In samples from days 35 and 49 PST, stained parasites were only observed intravascular, in the blood vessels or secondary lamellar lacunae (pillar-cell-delimited vascular space in the pseudobranch secondary lamellae). On day 35, only a few stained parasites were observed, mostly in the major vessels centrally in the filaments (Fig. 4a-c), but occasionally in the lamellar lacunae. Parasite stages could be observed at day 35 and 49 PST when comparing parallel sections stained with ISH and HE (Fig. 4b, c). A higher number of stained parasites were observed on day 49 compared to day 35, all vascular in the lamellar lacunae (Fig. 4d). The pseudobranch tissue showed normal structure in these samples (Fig. 4e). Samples at day 89 were characterized by a preponderance of extravascular stages in the pseudobranch (Fig. 4f, g), and areas with high numbers of parasite stages appeared irregular, disrupted, with high interlamellar cellularity but with few normal pseudobranch cells (Fig. 4h). However, moderate numbers of intravascular stages were seen in the gill arterioles at day 89 (Fig. 5a), the only observation of the parasite in the gills of the fish examined. At day 147 PST, the parasite was found to show a compartmentalized distribution in the pseudobranch. The basic gill like pseudobranch structure consists of primary lamellae, between facing rows of secondary lamellae. The parasite occurred in high numbers in some of these spaces between primary lamellae, while being comparatively rare in others (Fig. 5b). The compartments with high parasite numbers had few intact pseudobranch cells (Fig. 5d, e), while the lightly infected ones appeared normal or were slightly affected. Parasites were not seen in the day 216 fish (e.g. Fig. 5g), but the secondary lamellar parts of the pseudobranch often showed a disrupted structure (Fig. 5g, h).

Conventional histology performed on gill, eye, spleen, kidney, and pseudobranch tissues revealed *P. pseudobranchicola* myxospores to be present in pseudobranchs at 89 and 147 days post-sea-transfer only (Fig. 5e). When examining wet preparations of fresh or frozen pseudobranch tissue, few and apparently immature myxospores were seen at day 89, while apparently free and mature myxospores occurred among other developmental stages in such samples at days 106 and 147 days PST (Fig. 5f). At day 147, histology revealed that parts of the pseudobranchs also showed considerable infiltration of immune cells. At day 216 PST the studied pseudobranchs showed extensive tissue disruptions (Fig. 5h), but spores or recognizable myxosporean stages were not observed. No developmental stages of *P. pseudobranchicola* were found in the blood smears.

Morphological details were usually not apparent in the ISH stained parasites. However, the intravascular parasites occurred as single stained bodies or as ‘doublets’. These stages varied from apparently mono to binuclear, the larger ones also containing 3–4 additional dense bodies (Fig. 4c). Single stained bodies measured 3.7–7.2 (mean 5.0) μm in diameter (*n* = 30), the doublets reaching 12 μm in length. The intravascular stages seen were of similar size in the different samples (days 35–89 PST). Very few of these cells could be recognized in the HE sections.

The extravascular stages in the pseudobranch were first observed at day 89 PST. They then were rounded and measured 4.2–5.5 μm in diameter, or oval and reaching 7.5 μm in length. At 147 days PST multicellular stages were abundant, seen as aggregates of nuclei. Non-lamellar parts of the pseudobranchs showed degenerative changes associated with lipid deposition in the cells, but contained few parasites.

## Discussion

The farm site in western Finnmark, chosen for this study, has a history of recurring parvicapsulosis. The studied smolts were sea launched *medio* August, a period when the *P. pseudobranchicola* infection pressure in the region is high [9]. Indeed, all fish were infected in our first sample date 21 days after sea transfer. The high infection pressure in the sea is likely due to the presence of infective actinospores in the water. The only other possibility is that the myxosporean infection is spread directly among the individuals in a pen-population. Direct transmission may occur for some enteric *Enteromyxum* spp. in captive fish. These myxosporeans may be transmitted directly through presporogonic stages [10, 11]. A similar direct transmission by early stages from the pseudobranchs or gills has therefore been considered a possibility for *P. pseudobranchicola*. However, intraperitoneal injection experiments performed with pseudobranch homogenates has not successfully caused infection (unpublished observations).

A factor that could contribute to the development of parvicapsulosis in farmed salmon is concurrent infections with other salmon pathogens [29]. Screening of the smolt just prior to sea-transfer revealed fish weakly positive for IPNV, but the fish did not develop IPN after sea launching. However, the salmon in the farm experienced an early outbreak of tenacibaculosis, which could have affected the susceptibility of the smolts to *P. pseudobranchicola*. The tenacibaculosis was rapidly dealt with by culling all fish in the most affected cage and by treating the rest of the fish with antibiotics. This action stopped the increasing mortality and morbidity at the site and the disease had only a minor effect on the salmon in the study cage. The fact that no other pathogens were detected during the period with heavy infections with *P. pseudobranchicola* (November-January) suggests that the mortality peak seen then was mainly caused by parvicapsulosis.

Most current members of the parvicapsulid genera *Parvicapsula* and *Gadimyxa* infect the urinary system of marine and anadromous fish [13, 30], and one species, *Parvicapsula* sp., may cause a chronic proliferative nephritis and mortality in farmed coho salmon *Oncorhynchus kisutch* [31, 32]. That species, appearing very similar to *P. pseudobranchicola*, also infects the pseudobranchs of the host [33]. *Parvicapsula pseudobranchicola* sporogony is most often observed in the pseudobranchs of the salmonid hosts [2, 5, 8, 19, 24], but has also been seen in liver, kidney and gills [19, pers. obs.]. Since these organs are much larger than the pseudobranchs, they could potentially be responsible for a significant part of the total spore output. However, among the studied organs and tissues, only the pseudobranchs showed high densities of the parasite. The blood was positive, but never showed high parasite densities. While the density estimates obtained for the different tissues are not directly comparable, the low parasite RNA levels detected may simply be due to presence of parasites in the blood. The low signal seen in gill, kidney and liver samples, implies that these organs were not significantly affected by the parasite. At peak parasite densities in the pseudobranchs, densities in liver, bile and gallbladder were very low, observations incompatible with parasite sporogony in the liver and spore release via bile. Densities were also low in the kidney and urinary bladder, the latter could be high if spore-release occurred from the kidneys, including unstudied parts. Therefore, no evidence was seen suggesting significant *P. pseudobranchicola* development in the kidney. However, apparently high parasite densities occurred in some samples from the eyes, when compared to the background signal from the blood. Possibly, parasite stages occur in the choroidea of the eyes, where they could directly influence the organ. In addition, since the arterial blood supply to the eyes pass-through the pseudobranchs, organ damage could obstruct blood passage or alter the blood chemistry, perhaps affecting vision in heavily infected fish. The typical clinical signs associated with parvicapsulosis could well be explained by vision impairment or blindness [2, 19]. Hence, this represents an area that needs further study.

A complication with field experiments is the likely continuous exposure to the studied agent, possibly repeatedly causing infections. In the present case, the fish were likely immediately exposed to actinospores following sea-transfer, but this exposure may have continued during the autumn. The observed positive blood may be due to *P. pseudobranchicola* blood stages, possibly originating from actinospore-sporoplasms. However, it is noteworthy that all pseudobranchs were infected in the first sample (21 days PST), while the prevalence in blood then was only 57%. Also, ISH stained sections shows blood stages that appear to adhere to the vascular endothelium in the pseudobranch or gills, and if the gills are ports of entry, the seeding of the pseudobranch with such stages could be very fast. The present study does not provide proof for any proliferative blood-stage in *P. pseudobranchicola*, since a continuous reinfection from the environment is also a possibility. The occurrence of the intravascular parasite stages in the pseudobranchs also appeared randomly distributed, an observation also suggesting that blood-stage division there may be limited or absent. However, the intravascular stages seen by ISH appeared to consist of single cells or ‘doublets’, similar to those observed in the related parvicapsulid *Gadimyxa atlantica*, in the glomeruli of cod (*Gadus morhua*) [17]. Possibly, these doublets are dividing or recently divided stages, causing a modest intravascular propagation of the parasite. At day 89 (November) however, the parasite stages in the pseudobranch were seen to be extravascular, and may have moved through the lacunar epithelia in a manner similar to leucocyte emigration (diapedesis). The extravascular appearance of the parasite was associated with an apparent pseudobranch-cell depletion, i.e. few intact cells were left [19]. This may also be due to an invasion and intracellular development of the parasite stages in these cells [3], so they are not recognizable. At day 147, the parasites in the pseudobranch showed a markedly compartmentalized occurrence, with high densities in some spaces between primary lamellae and few in others. This is an observation suggestive of parasite proliferation in these compartments, preceding sporogony. This observation also suggests that in limited infections, normal pseudobranch function may be sustained by unaffected parts of the organ.

In the present study, we expected the development of clinical parvicapsulosis. This was observed, but was limited and the associated mortality low. Since we aimed at getting random fish from the cage, few of these may have been significantly affected by the parasite. A higher prevalence of runting, higher mortality and more extensive pseudobranch infections and lesions have been seen in other outbreaks [2, 19, 23], and the more serious clinical picture associated with heavy infections could be due to pseudobranch dysfunction. The role of this organ in salmonids is poorly known, but several lines of evidence from different teleosts suggest a role in preconditioning of afferent blood to the eyes [22, 34, 35].

The limited increased mortality observed that was not related to the early tenacibaculosis outbreak occurred during winter (December 2014 to January 2015). Parasite spores were first observed in November (day 89), but clearly fully mature spores occurred in late November (day 106) and in January (day 147). No spores were seen in March. This is at variance with some previous studies, recording spores throughout spring until June. However, in those cases the fish had been put to sea later in autumn [8], and the timing of the first infections were not examined. The present study indicates that sporogony may occur within 3–5 months, representing some 720–1200 day-degrees. Between our January and March samples (days 147 and 216), a marked decrease was seen in the parasite density in the pseudobranchs. This coincided with the disappearance of the mature spores. An extensive tissue disruption in the organ was seen histologically at day 216, likely due to the spore release. March is therefore a period when mature spores from autumn stocked farmed Atlantic salmon may be released to the environment. However, this could be an anomaly to the parasite, since wild anadromous salmonids in northern Norway move to sea earlier than August, in June–July (e.g. [36]). Spring stocked farmed salmon in northern Norway may harbour spores after 4 months, e.g. fish put to sea in early May harbour spores by 1st September [8]. Therefore, the spore release from wild salmonids as well as spring-stocked farmed salmon may occur during autumn. This may be a ‘natural’ period for the annelid alternate host to become infected by myxospores.

After the marked March decline in parasite densities in the pseudobranchs, there was a further gradual decline when we followed the salmon through nearly two years in the sea. During the second autumn in the sea, the salmon were undoubtedly again exposed to actinospores. However, there was no indication of increasing parasite densities in the salmon the second autumn-winter. This observation therefore represents evidence for acquired immunity to this myxosporean in salmon, also supported by field-experience suggesting that this infection is a problem mainly the first year at sea. Other parvicapsulids, such as *P. minibicornis* and *Gadimyxa atlantica* may infect both juvenile and adult fish. In the case of *G. atlantica*, blood was found to be positive for the parasite in the 0-group cod and not in the 1-group fish [17]. However, larger cod are commonly infected, so they are either repeatedly infected from the environment, or the infections are persistent [13, 17, 37]. *Parvicapsula minibicornis* infections contracted by Pacific salmon juveniles in freshwater could be cleared in the marine phase, since returning adults are PCR negative for the parasite when entering the rivers [38]. Since prevalence in adult fish at the spawning grounds may be high [38, 39], protective immunity in adults from past infections seem unlikely.

## Conclusions

Salmon rapidly became infected with *P. pseudobranchicola* after sea transfer in August, and then parasite densities in the pseudobranchs peaked in winter. Mature spores first appeared in November, and were mainly released in the period January-March, when parasite densities decreased. Clinical signs of parvicapsulosis were associated with high parasite densities in the pseudobranchs. Atlantic salmon appear to develop immunity to the parasite as shown by the fact that parasite density did not increase during the second year in the sea. The main site of the parasite in Atlantic salmon was found to be the pseudobranchs, most other tissues were positive but showed low parasite (RNA) densities. The choroidea of the eyes could be an exception, and the influence of the parasite on the eyes needs further study. Future research should focus on identifying the marine polychaete alternate host of *P. pseudobranchicola*. Actinospores from infected worms could allow controlled challenge experiments, promoting studies improving both our understanding of the disease, and the prophylaxis and control of the infections.

## Additional file

Additional file 1: Table S1. Dataset. (XLSX 117 kb)

## Abbreviations

HE: Hematoxylin and eosin
ISH: *in situ* hybridization
NE: Normalized expression
PST: Post-sea-transfer
RT-PCR: Reverse-transcription PCR
